# Improvements in extraction yield by solid phase lipid extraction from liquid infant formula and human milk, and the fatty acid distribution in milk TAG analyzed by joint JOCS/AOCS official method Ch 3a-19

**DOI:** 10.3389/fnut.2022.970837

**Published:** 2022-09-16

**Authors:** Yomi Watanabe, Motoko Hirokawa, Arisa Furukawa, Lisa Kawasaki, Hiroko Takumi, Araki Masuyama

**Affiliations:** ^1^Research Division of Biomaterials and Commodity Chemicals, Osaka Research Institute of Industrial Science and Technology, Osaka, Japan; ^2^Applied Research Laboratory, Ezaki Glico Co., Ltd., Osaka, Japan; ^3^Department of Applied Chemistry, Osaka Institute of Technology, Osaka, Japan

**Keywords:** Röse-Gottlieb extraction, human breast milk, solid phase extraction (SPE), ruminant milk fat, TAG, positional distribution of fatty acids

## Abstract

The Röse-Gottlieb method is one of the most widely used methods for extracting lipids from milk samples. However, we found that lipid recovery from liquid infant formula and human breast milk was lower than expected. Better lipid recovery from these liquid matrices was obtained by solid phase extraction using silica gel; ~10% more could be recovered from liquid infant formula and ruminant milk, and 25% more from human breast milk. However, the method is not recommended for lipid extraction from dried whole milk powders.

## Introduction

The nutritional value of infant formulas is becoming increasingly important as the equalization of social and household roles of mothers and fathers progresses. The decrease in the number of babies per couple might also lead parents to prefer infant formula of better quality and safety. Manufacturers of infant formula are constantly striving to match the nutritional value of human milk, including lipids. Human milk lipid is unique in that the structure of the triacylglycerols is largely symmetrical, with oleic acid in the *sn*-1 and -3 positions, and palmitic acid in the *sn*-2 position (1, 2) (OPO, 18:1-16:0-18:1). We recently compared the lipid composition and structure of several infant formulas in the Japanese market to human milk after extracting lipids from the infant formula samples by the Röse-Gottlieb method (3). The Röse-Gottlieb method (4, 5) is one of the most common methods for extracting neutral lipids from dried milk products. In our hands, 92–96% of the lipids could be extracted from most infant formula samples. However, <80% of the lipid could be extracted from a liquid formula (3). Improvement in yield was not obtained by increasing the time and temperature of sample heating with ammonia water prior to solvent extraction, by increasing the volume of extraction solvent per volume infant formula, or by using chloroform or dichloromethane as an extraction solvent. The chloroform/methanol extraction of milk samples increased gravimetrical yield than Röse-Gottlieb method. However, the increased matter was found not to be ether soluble, and was indicated to be carbohydrates mainly by IR spectrum. We postulated chloroform/methanol extracted some glycolipids from milk. The target of this study is neutral lipids, TAG, in milk. Thus, chloroform/MeOH was not desirable as the extraction solvent this time. This study presents that solid phase extraction using silica gel resulted in an improvement in neutral lipid recovery from both liquid infant formula and ruminant milk of ~10% over the Röse-Gottlieb method. Solid phase extraction was even more effective in extracting lipids from human milk, increasing the lipid recovery more than 25%.

## Materials and methods

### Materials

Liquid infant formula, goat and cow milk were obtained in the local market. Dried cow milk powder was from Yotsuba Milk Product Co. (Sapporo, Japan). Dried goat milk powder was purchased from Shinko Kikaku Co. (Yokohama, Japan, produced in the Netherlands) and dried camel milk powder was from Aadvik Foods and Products Private Co. (Nokha, India). Human breast milk samples, each from a single donor, were purchased from Lee Biosolutions, Inc. (Maryland Heights, MO, USA). Their lipid contents were determined using a Miris Human Milk Analyzer (Uppsala, Sweden). Milk samples were divided into 2 groups and mixed to prepare ~25 ml each of high- and low-fat human milk. Immobilized *Candida antarctica* lipase B preparation, lipase CL “Amano” IM, was provided by Amano Enzyme (Gifu, Japan). Inert-Sep-SI silica gel columns (0.69 g) were from GL Science (Tokyo, Japan). Ethanol was dried over molecular sieves 3A. Silica gel 60, ethanol, methanol, hexane, diethyl ether, potassium hydroxide, and other reagents were purchased from FUJIFILM Wako Pure Chemical Corp. (Tokyo, Japan). Reagents were of analytical grade.

### Extraction of lipids by Röse-Gottlieb method

Lipids in milk and liquid infant formula were extracted by the Röse-Gottlieb method as described in AOAC Official Method 932.02 (4, 5). In brief, 10 ml milk was mixed with 1.5 ml of 28% ammonia water, and the mixture was incubated at 65 ± 5°C for 15 min with occasional mixing. To the solution, 10 ml of ethanol and several drops of 1% phenolphthalein/ethanolic solution were added, and this mixture was vigorously shaken with 25 ml of diethyl ether. Petroleum ether (25 ml) was added, and shaking was repeated. The ether layer was recovered after phase separation. An additional 4 ml of ethanol was added to the water layer, which was then extracted with 15 ml diethyl ether followed by extracting with 15 ml petroleum ether. This extraction step was repeated once more. All the ether layers were combined and dried over anhydrous sodium sulfate. The solvent was removed under reduced pressure until the weight of the extracts reached to give the constant values. Extractions were conducted twice, and mean values are presented.

### Extraction of lipids by silica gel

Silica gel 60 and liquid infant formula or milk (10 ml) were added to a 300 ml flask and mixed. The suspension was evaporated using a rotary evaporator at 120 hPa and 50°C initially, gradually decreasing pressure to 15 hPa and increasing water-bath temperature to 80°C. Evaporation was occasionally stopped, and lumps of milk-adsorbed silica gel was crushed with a spoon into smaller pieces to assist in removing water. Drying continued until the silica gel/milk texture was powdery again. The silica gel/milk powder was loaded into a glass chromatography column with a diameter of 30 mm, and lipid was eluted with 100~200 ml of diethyl ether: petroleum ether (1:1, v/v). For human breast milk, 15 g silica gel was mixed with 5 ml milk, dried, and eluted with 100 ml solvent. The solvent was removed from the eluents under reduced pressure to obtain the recovered lipids.

### Lipid content in goat milk

The goat milk was freeze-dried (FDU-1110, EYELA, Tokyo, Japan), and crushed to obtain a dried goat milk powder. A fixed amount of the internal standard, undecanoic acid methyl ester, was added to the dried milk powder and methyl esters were prepared by 0.5 M potassium hydroxide methanolic solution. Lipid amounts were calculated based on the peak areas of the internal standard and of other fatty acid methyl esters.

### Fatty acid distribution analysis by joint JOCS/AOCS official method Ch 3a-19

Fatty acid distribution of lipids were analyzed according to Joint JOCS/AOCS official method Ch 3a-19 (6). Extracted lipids (0.25 g), dehydrated ethanol (2.5 g), and Lipase CL “Amano” IM (0.10 g) were added to a glass vial and shaken at 30°C for 3 h. The reaction mixtures were filtered through absorbent cotton to remove immobilized lipase, transferred to a new glass tube or evaporation flask, and stored at −20°C until further use.

Ethanol in the 2 ml reaction mixtures was removed with a rotary evaporator. The resulting oil (0.1 ml) was immediately loaded onto an Inert-Sep-SI SPE column, pre-equilibrated with 10 ml hexane:diethyl ether, 8:2 (v/v). The column was eluted with 10 ml of the same solvent mixture to obtain fraction 1, consisting mainly of fatty acid ethyl esters (FAEEs); then rinsed with 20 ml of the same solvent to flush out diacylglycerols (DAGs). Finally, the column was eluted with 10 ml diethyl ether to obtain fraction 2, which consisted mainly of *sn*-2 MAG. This fraction was brought to dryness under reduced pressure and methylated using 0.5 M potassium hydroxide methanolic solution.

The methylated samples were analyzed by GC (Agilent Technologies, Santa Clara, USA) equipped with a DB-23 column (0.25 mm, 0.25 μm, 30.0 m, Agilent Technologies). The column temperature was controlled at 60°C for 3 min, raised to 180°C at the rate of 8.0°C/min, and raised to 240°C at the rate of 3.0°C/min. Injector and detector (FID) temperatures were set at 245 and 250°C, respectively. All analyses were carried out twice and the mean values are presented.

The FID responses of the GC were corrected to the molar ratios by the theoretical FID response factors according to Joint JOCS/AOCS official method Ch 3a-19 (6, 7). Briefly, the area percent of each FA alkyl ester was divided by the respective active carbon numbers (carbon number of each FA alkyl esters−1) and by the atomic weight of carbon (12.01) to obtain the molar ratios of FA.

The molar ratio of each fatty acid (MX) is calculated as follows:

*Mx* = *A*/ [*ACN* × *AW*_C_]

where

*Mx* is the molar ratio of each fatty acid methyl ester;

A is the area percentage of fatty acid methyl ester;

ACN is the Active Carbon Number (Carbon number of each fatty acid methyl ester minus 1);

AW_C_ is the atomic weight of carbon (12.01).

The percentage by mole of each fatty acid (Y) is calculated as follows:

*Y* = *M*_x_/*M*_T_ ×100

where

Y is the molar percentage of each fatty acid;

M_X_ is the molar ratio of each fatty acid methyl ester

M_T_ is the sum of M_X_.

## Results

### Solid phase extraction of lipid from ruminant milk and liquid infant formula

Initial experiments establishing the conditions required for lipid extraction were carried out with liquid goat milk. Goat milk (10 ml; lipid content, 2.8%) was added to 5~15 g silica gel, mixed, and water was removed under reduced pressure using a rotary evaporator. Lipids were eluted from the dried silica gel by a mixture of diethyl ether and petroleum ether (1:1, v/v). The recovered lipid was weighed after the removal of the solvent. The recovery was calculated by comparing the amounts extracted with the theoretical amounts. The recovery of lipids from goat milk by solid phase extraction reached *ca*. 0.26 g/10 ml milk, which was an increase in the yield of ~10% compared to the Röse-Gottlieb method (*ca*. 0.23 g/10 ml milk, Table 1).

**Table 1:** Effect of the amount of silica gel on lipid recovery.

	**Lipid content^a^(%)**	**Silica gel (g/ml milk)**	**Lipid yield (g/10 ml milk)**
Goat milk	2.9^b^	RG^c^	0.225 ± 0.013
		0.5	0.253 ± 0.018
		1	0.266 ± 0.018
		1.5	0.254 ± 0.019
Cow milk	4.3	RG^c^	0.381 ± 0.005
		0.5	0.359 ± 0.001
		1	0.419 ± 0.015
		1.5	0.423 ± 0.021
		2	0.401 ± 0.034
Liquid	3.8	RG^c^	0.277 ± 0.008
Infant formula		1	0.287 ± 0.023
		1.5	0.295 ± 0.014
		2	0.312 ± 0.009
Cream	47	2	4.41 ± 0.424
(Cow milk)		3	4.63 ± 0.311
		4	4.37 ± 0.417

^*a*^The lipid content displayed on the product label. ^*b*^The lipid content was determined as described in Materials and Method Section Lipid content in goat milk. ^*c*^Röse-Gottlieb extraction method.

The recovery from cow milk (lipid content, 4.3%) was also improved to 0.42 g/10 ml milk by using 1~2 g silica gel/ml milk from 0.38 g/10 ml milk by the Röse-Gottlieb method (Table 1), with the improved yield of *ca*. 10% (Supplementary Figure 1). Then, the lipid in the liquid infant formula (lipid content, 3.8%) was extracted by SPE. The recovery reached 0.31 g/10 ml milk when 2 g silica gel was used per 1 ml milk, whereas it was 0.28 g/10 ml milk by the Röse-Gottlieb method (Table 1) The extracted lipid was predominantly TAG when analyzed by TLC and TLC-FID (data not shown). The fatty acid compositions of the liquid infant formula extracts obtained by both methods were similar (Supplementary Table 1). When solid phase extraction was applied to a cream of cow milk with lipid content of 47%, 4.4~4.6 g/10 ml milk was achieved with 2~4 g silica gel/ml cream (Table 1). It was unfortunate that the appropriate amount of silica gel to a volume of milk could not exactly be fixed due to the experimental error. However, we estimate that silica gel in a range of 1~2 g/ml milk would be good.

### Solid phase extraction of lipid from reconstituted dried ruminant milk

Commercially dried goat milk powder (1 g, lipid content 15%) was dissolved in 8.5 ml 40°C water, 10 g silica gel was added, and drying and elution with 200 ml diethyl ether:petroleum ether (1:1, v/v) was carried out. However, no lipid was obtained. From 1 g of commercially dried whole cow milk powder (lipid content, 30%), only 0.21 ± 0.002 g lipid was recovered where 0.3 g was expected. From commercially dried camel milk (lipid content 33%) adsorbed on 30 g silica gel, only 0.22 ± 0.02 g oil was obtained where 0.33 g was expected. Thus, the lipid recoveries were 0~66% from the dried ruminant milk powders. Thus, optimization of the solid phase extraction will be required for dried milk powders.

### Solid phase extraction of lipid from human breast milk

When the Röse-Gottlieb extraction method was applied to human breast milk, ~60% lipid recovery was obtained, as indicated by the Miris human milk analyzer (Table 2). When solid phase extraction using 3 g silica gel/ml was applied to human milk samples, the recovery from high fat milk samples (lipid content, 4.5%), improved from 62% obtained by the Röse-Gottlieb method to 89% by SPE (Table 2). From low fat milk sample (lipid content, 1.89%), solid phase extraction achieved 85% recovery. Thus, lipid recovery from human breast milk was increased more than 25% using solid phase extraction.

**Table 2:** Röse-Gottlieb and solid phase extraction of lipids from human breast milk.

**Extraction method**	**Recovery (%)**	
	**High fat milk**	**Low fat milk**
Solid phase	85.1 ± 5.7	89.0 ± 7.9
Röse-Gottlieb	60.3 ± 2.7	61.5 ± 6.4

The lipid contents of the high fat milk was 4.54 g/100 ml, and that of the low fat milk was 1.89 g/100 ml.

The fatty acid compositions of lipids extracted by the two methods from both high- and low-fat human breast milk were similar (Table 3). The major FAs were 16:0, 18:1, and 18:2.

**Table 3:** FA composition of lipid (TAG) extracted by SPE or Röse-Gottlieb method from human milk.

**Fatty acid**	**Composition (mol%), high fat milk**		**Composition (mol%), low fat milk**	
	**SPE**	**Röse-Gottlieb**	**SPE**	**Röse-Gottlieb**
6:0	0.82 ± 0.32	0.95 ± 0.18	4.10 ± 0.64	5.94 ± 0.48
8:0	0.93 ± 0.01	1.21 ± 0.08	1.06 ± 0.00	0.44 ± 0.01
10:0	1.08 ± 0.02	1.19 ± 0.05	1.18 ± 0.01	1.49 ± 0.05
10:1	0.73 ± 0.01	0.90 ± 0.05	0.78 ± 0.02	0.34 ± 0.02
12:0	5.78 ± 0.06	5.96 ± 0.03	5.45 ± 0.06	5.91 ± 0.10
12:1t	0.49 ± 0.08	0.56 ± 0.09	0.56 ± 0.00	0.26 ± 0.01
12:1	0.49 ± 0.02	0.60 ± 0.01	0.56 ± 0.01	0.13 ± 0.19
14:0	6.68 ± 0.03	6.70 ± 0.02	7.23 ± 1.62	6.38 ± 0.05
15:0	0.27 ± 0.00	0.26 ± 0.01	0.13 ± 0.18	0.31 ± 0.01
16:0	26.55 ± 0.01	26.19 ± 0.10	25.77 ± 0.18	24.80 ± 0.07
16:1	2.23 ± 0.02	2.26 ± 0.09	1.88 ± 0.29	2.01 ± 0.07
17:0	0.23 ± 0.01	0.24 ± 0.00	0.30 ± 0.04	0.30 ± 0.00
17:1	0.11 ± 0.02	0.11 ± 0.00	0.13 ± 0.02	0.12 ± 0.01
18:0	6.72 ± 0.05	6.64 ± 0.00	6.49 ± 0.07	6.37 ± 0.03
18:1	31.14 ± 0.35	30.99 ± 0.04	29.40 ± 0.23	29.70 ± 0.06
18:2 n-6	13.02 ± 0.05	12.89 ± 0.02	13.03 ± 0.09	13.32 ± 0.06
18:3 n-3	0.82 ± 0.04	0.77 ± 0.02	0.82 ± 0.02	0.89 ± 0.00
CLA	0.34 ± 0.04	0.28 ± 0.02	0.16 ± 0.22	0.20 ± 0.02
20:0	0.28 ± 0.02	0.28 ± 0.03	0.14 ± 0.19	0.17 ± 0.01
20:1	0.41 ± 0.22	0.26 ± 0.03	0.14 ± 0.20	0.29 ± 0.01
others	0.88 ± 0.29	0.76 ± 0.02	0.71 ± 0.44	0.62 ± 0.15
Sum	100	100	100	100

### Fatty acid distribution analysis of human breast milk by joint JOCS/AOCS official method Ch 3a-19

As sufficient amounts of lipids were extracted from high-fat human breast milk, its fatty acid distribution was analyzed by Joint JOCS/AOCS Official Method Ch 3a-19 (Table 4). The FA compositions of human milk lipids at the *sn*-2 position extracted by both methods were similar. The molar distribution of each FA at the *sn*-1(3) and *sn*-2 positions is also shown in Table 3. Of the total 16:0 in TAG, 70% was located at *sn*-2, and 30% was at *sn*-1(3). As for 18:1, 90% was located at *sn*-1(3) and 10% at *sn*-2. These observations agreed well with previous reports (1, 2). Here, it was confirmed that SPE increased the lipid recovery compared to the conventional Röse-Gottlieb method without affecting total FA composition of the extracts (Table 3) and the FA distribution as determined by Joint JOCS/AOCS Official Method Ch 3a-19 (Table 4).

**Table 4:** FA composition at *sn*-2 and FA distribution in high fat milk analyzed by Joint JOCS/AOCS Official Method Ch 3a-19.

	**SPE extraction**		**Röse-Gottlieb extraction**	
**Fatty acid**	**Composition (mol%)**	**Distribution**	**Composition (mol%)**	**Distribution**
	***sn*-2^a^**	**1,3^b^ : 2^c^**	***sn*-2^a^**	**1,3^b^ : 2^c^**
6:0	2.48 ± 0.11	−1 : 101	2.87 ± 2.23	−1 : 101
8:0	0.25 ± 0.33	91 : 9	−	100 : 0
10:0	0.61 ± 0.01	81 : 19	0.56 ± 0.06	84 : 16
10:1	−	100 : 0	−	100 : 0
12:0	7.03 ± 0.08	59 : 41	6.92 ± 0.20	61 : 39
12:1t	0.24 ± 0.05	83 : 17	0.41 ± 0.12	76 : 24
12:1	0.14 ± 0.18	91 : 9	0.16 ± 0.22	91 : 9
14:0	11.94 ± 0.17	40 : 60	12.19 ± 0.48	39 : 61
15:0	0.57 ± 0.02	31 : 69	0.57 ± 0.02	28 : 72
16:0	55.65 ± 0.72	30 : 70	55.97 ± 0.73	29 : 71
16:1	2.00 ± 0.04	70 : 30	2.06 ± 0.05	70 : 30
17:0	0.22 ± 0.00	67 : 33	0.24 ± 0.04	67 : 33
17:1	0.12 ± 0.00	63 : 37	0.13 ± 0.02	61 : 39
18:0	1.26 ± 0.01	94 : 6	1.07 ± 0.19	95 : 5
18:1	8.59 ± 0.13	91 : 9	8.31 ± 0.69	91 : 9
18:2 n-6	6.37 ± 0.05	84 : 16	6.14 ± 0.17	84 : 16
18:3 n-3	0.44 ± 0.01	82 : 18	0.59 ± 0.25	75 : 25
CLA	0.14 ± 0.01	86 : 14	0.14 ± 0.00	83 : 17
20:0	0.15 ± 0.01	82 : 18	0.15 ± 0.00	82 : 18
20:1	0.33 ± 0.17	73 : 27	0.18 ± 0.00	77 : 23
others	1.45 ± 0.48	45 : 55	1.34 ± 0.68	41 : 59
Sum	100	67 : 33	100	67 : 33

^*a*^The value is expressed as β. α= [TAG in mol% (Table 3)] x 3 - β.

^*b*^100 x α / (α + β).

^*c*^100 x β / (α + β).

## Discussion

Liquid infant formula is a drink-ready product for babies, freeing parents and caretakers from preparing hot water, measuring and dissolving the proper amount of powdered infant formula, and cooling the prepared formula to drinkable temperature, while the baby is crying for milk. The product is especially beneficial under the circumstances of natural disaster, where the supply of drinking water and electricity are limited. Liquid infant formula is highly homogenized to prevent phase separation during storage and re-warming and provide long shelf-life. This stable homogeneous state might reduce lipid recovery when extracted by the conventional Röse-Gottlieb method (3). We postulated that silica gel may help disrupt the emulsion of the formula and improve the recovery of the lipid. We postulated this based on the observation that during liquid-liquid extraction in the Röse-Gottlieb method, especially at the second and third extraction step, emulsions often form in the organic phase even in the presence of ethanol.

In the Röse-Gottlieb method, milk is treated with ammonium water for 15 min at 65°C prior to the liquid-liquid extraction step to disrupt the stable assembly of milk protein and lipid. This step was not needed in the SPE method, as the recovery was not changed with or without treatment. However, the step of dehydrating silica gel after the adsorption of milk could not be omitted. We postulate that the presence of water might inhibit the adsorption of milk lipid to silica gel, and/or interfere with contact between elution solvent and milk-absorbed silica gel.

The increase in the recovery by the SPE method compared to the conventional Röse-Gottlieb method was *ca*. 10% from liquid infant formula and ruminant milk and was 25% from human breast milk. In cases where recovery by the Röse-Gottlieb method is lower than expected, the SPE method would be worth trying.

## Data availability statement

The original contributions presented in the study are included in the article/Supplementary material, further inquiries can be directed to the corresponding author.

## Author contributions

YW conceived and designed the study, carried out the research, and wrote the first draft of the manuscript. MH, AF, and LK carried out the research. HT and AM provided valuable resources in support of this research. All authors contributed to the article and approved the final draft of the manuscript.

## Conflict of interest

Authors MH and HT are employed by Ezaki Glico Co., Ltd.

The remaining authors declare that the research was conducted in the absence of any commercial or financial relationships that could be construed as a potential conflict of interest.

## Publisher's note

All claims expressed in this article are solely those of the authors and do not necessarily represent those of their affiliated organizations, or those of the publisher, the editors and the reviewers. Any product that may be evaluated in this article, or claim that may be made by its manufacturer, is not guaranteed or endorsed by the publisher.

## References

[1] Giuffrida F Marmet C Tavazzi I Fontannnaz P Sauser J Lee LY. Quantification of 1,3-olein-2-palmitin (OPO) and palmitic acid in *sn*-2 position of triacylglycerols in human milk by liquid chromatography coupled with mass spectrometry. Molecules. (2019) 24:22. 10.3390/molecules24010022 30577597 PMC6337272

[2] López-López A López-Sabater MC Campoy-Folgoso C Rivero-Urgell M Castellote-Bargalló AI. Fatty acid and *sn*-2 fatty acid composition in human milk from Granada (Spain) and in infant formulas. Eur J Clin Nutr. (2002) 56:1242–54. 10.1038/sj.ejcn.1601470 12494309

[3] Watanabe Y Kawasaki L Masuyama A. Analysis of fatty acid distribution in lipid extracts of infant formulas. Milk Science. (2020) 69:63–70. 10.11465/milk.69.63

[4] AOAC Official Method 932.02-1945. Fat (Crude) or Ether Extract in Dried Milk Products. Rockville, MD: AOAC International.

[5] Suzuki C Yasuda S. Study of crude fat measurement methods in dried whole milk and formula feed using it as a main ingredient. Research Report of Animal Feed. (2018) 43:17–21. Available online at: http://www.famic.go.jp/ffis/feed/obj/rraf43-03.pdf

[6] Joint JOCS/AOCS Official Method Ch 3a-19. Determination of the composition of fatty acids at the 2-position of oils and fats-enzymatic transesterification method using Candida antarctica lipase. Urbana, IL: AOCS.

[7] Yoshinaga K Sato S Sasaki R Asada M Hori R Imagi J. The collaborative study on the enzymatic analysis of positional distribution of short- and medium chain fatty acids in milk fat using immobilized *Candida antarctica* lipase *B J Oleo Sci*. (2016) 65:291–302. 10.5650/jos.ess15260 26972465

