# Supplementary material for: Improvements in extraction yield by solid phase lipid extraction from liquid infant formula and human milk, and the fatty acid distribution in milk TAG analyzed by joint JOCS/AOCS official method Ch 3a-19
Source: Front Nutr. 2022 Sep 16;9:970837. doi: 10.3389/fnut.2022.970837 (PMC9523589; doi:10.3389/fnut.2022.970837)
Supplement: Supplementary file 1 [file Table_1.docx]

Supplementary Table 1. FA composition of TAG extracted by SPE or the Röse-Gottlieb method from a liquid infant formula. An average of n2 was presented.

| Fatty acid | Composition (mol%) | |
| --- | --- | --- |
|  | SPE | Röse-Gottlieb |
| 4:0 | 0.07 ± 0.00 | 0.07 ± 0.18 |
| 6:0 | 0.78 ± 0.01 | 0.80 ± 0.00 |
| 8:0 | 1.91 ± 0.01 | 1.87 ± 0.00 |
| 10:0 | 1.29 ± 0.00 | 1.28 ± 0.02 |
| 12:0 | 9.44 ± 0.03 | 9.43 ± 0.02 |
| 14:0 | 4.48 ± 0.01 | 4.48 ± 0.01 |
| 16:0 | 22.16 ± 0.03 | 22.27 ± 0.02 |
| 18:0 | 8.01 ± 0.01 | 8.03 ± 0.00 |
| 18:1 | 33.78 ± 0.03 | 33.94 ± 0.01 |
| 18:2 n-6 | 12.86 ± 0.01 | 12.71 ± 0.03 |
| 18:3 n-3 | 2.02 ± 0.01 | 1.91 ± 0.00 |
| CLA | 0.14 ± 0.00 | 0.14 ± 0.00 |
| others | 3.07 | 3.08 |
| sum | 100 | 100 |
