# Supplementary material for: Improvements in extraction yield by solid phase lipid extraction from liquid infant formula and human milk, and the fatty acid distribution in milk TAG analyzed by joint JOCS/AOCS official method Ch 3a-19
Source: Front Nutr. 2022 Sep 16;9:970837. doi: 10.3389/fnut.2022.970837 (PMC9523589; doi:10.3389/fnut.2022.970837)
Supplement: Supplementary file 2 [file Image_1.pdf]

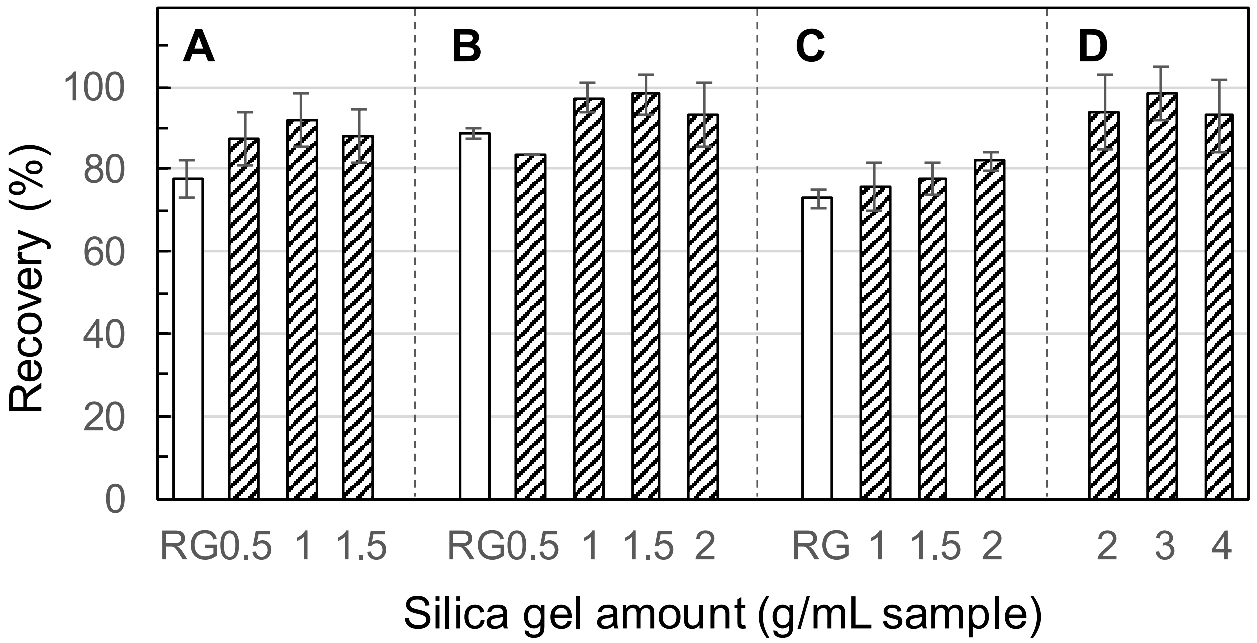

Supplementary Fig. 1 Effect of the amount of silica gel on lipid recovery. Open bars, the recovery of lipid extracted by the Röse-Gottlieb method (RG); striped bars, recovery by solid phase extraction. The recovery was expressed relative to the lipid content displayed on the product labels, except goat milk. The lipid content in goat milk was determined as described in Materials and Method section 2.4. A, Goat milk (lipid content, 2.8%), B, high fat cow milk (4.3%), C, liquid infant formula (3.8%), D, cream from cow milk (47%).
